# Attentional, emotional, and behavioral response toward spiders, scorpions, crabs, and snakes provides no evidence for generalized fear between spiders and scorpions

**DOI:** 10.1038/s41598-023-48229-8

**Published:** 2023-11-28

**Authors:** E. Landová, I. Štolhoferová, B. Vobrubová, J. Polák, K. Sedláčková, M. Janovcová, S. Rádlová, D. Frynta

**Affiliations:** 1 Department of Zoology, Faculty of Science, Charles University, Prague, Czech Republic; 2 National Institute of Mental Health, Klecany, Czech Republic

**Keywords:** Psychology, Human behaviour

## Abstract

Spiders are among the animals evoking the highest fear and disgust and such a complex response might have been formed throughout human evolution. Ironically, most spiders do not present a serious threat, so the evolutionary explanation remains questionable. We suggest that other chelicerates, such as scorpions, have been potentially important in the formation and fixation of the spider-like category. In this eye-tracking study, we focused on the attentional, behavioral, and emotional response to images of spiders, scorpions, snakes, and crabs used as task-irrelevant distractors. Results show that spider-fearful subjects were selectively distracted by images of spiders and crabs. Interestingly, these stimuli were not rated as eliciting high fear contrary to the other animals. We hypothesize that spider-fearful participants might have mistaken crabs for spiders based on their shared physical characteristics. In contrast, subjects with no fear of spiders were the most distracted by snakes and scorpions which supports the view that scorpions as well as snakes are prioritized evolutionary relevant stimuli. We also found that the reaction time increased systematically with increasing subjective fear of spiders only when using spiders (and crabs to some extent) but not snakes and scorpions as distractors. The maximal pupil response covered not only the attentional and cognitive response but was also tightly correlated with the fear ratings of the picture stimuli. However, participants’ fear of spiders did not affect individual reactions to scorpions measured by the maximal pupil response. We conclude that scorpions are evolutionary fear-relevant stimuli, however, the generalization between scorpions and spiders was not supported in spider-fearful participants. This result might be important for a better understanding of the evolution of spider phobia.

## Introduction

Throughout our evolutionary past, humans have developed several complex adaptations about how to respond to life-threatening stimuli such as various predators^1^, venomous snakes^2^, or enraged conspecifics^3^. Early detection of these ancestral dangers is often accompanied by a strong emotional response, which further affects the following conscious attention and rapid adaptive behavioral response^4, 5^. Evolutionary relevant threatening animal stimuli are thought to activate the fear module^6^, a complex biopsychological system whose concept has been derived from the preparedness theory proposed by Seligman^7^ and elaborated by Mineka and Öhman^8^. Preparedness theory suggests that quick fear learning and its slow extinction are predominantly associated with those stimuli that have posed a threat to human ancestors. Numerous studies built upon the preparedness theory and fear module, testing their assumptions^9–11^. Here, we focus on how fear-relevant animals affect attention during an animal-unrelated task.

By far, the most often investigated animal in fear module research is the snake. In her snake detection theory, Isbell^12^ suggested that the evolution of the primate visual system has been strongly shaped by the need for rapid detection of snakes. A large body of evidence has been found in support of this hypothesis^13–16^, although some discussion is being held on its ecological validity^17^. Several studies reported specific early as well as late attentional changes in brain activity in response to snakes^18–20^. Snakes were also shown to be detected faster than other animals in visual search tasks using an eye-tracking camera^21, 22^, even in suboptimal visual conditions^23^. Venomous snakes, especially viperids, also evoked elevated psychophysiological responses^24^. Recently, the coevolution between snakes and primates was illustrated in an example of snake venom and resistance to it in primates^25^. Altogether, common fear and negative attitude toward snakes seem evolutionary well based^26^.

Contrary to that, the origin of spider fear remains uncertain despite its relatively large prevalence in the general population. High and exaggerated fear of spiders, arachnophobia, is one of the most common anxiety disorders with a prevalence of 2.7–6.1%^27, 28^. Most spider species have not been seriously dangerous for contemporary humans or their ancestors [Ref.^29^, reviewed in Ref.^30^] and thus, the evolutionary explanation of arachnophobia is questionable. Some authors suggest that the fear of spiders is driven by contamination-based disgust (reviewed in^28^). Matchett and Davey^31^ proposed the disease-avoidance model hypothesizing that spider phobia stems from the disgusting properties of the spider^32, 33^ and fear of involuntary physical contact with it. Davey^34^ suggested that the spider’s disgust-relevant properties had become apparent during the plague pandemics in the Middle Ages. As the disease etiology remained unknown, spiders served as a displaced target.

In some studies, spiders are viewed as prototypical fear stimuli similar to snakes^2, 35^, but event-related potential studies comparing attention to spiders, snakes, and other animals showed that the brain potential related to exogenous attention (P1) had the highest amplitude in response to snakes but not spiders, while enhanced attention-related brain activity (LPP) was found in response to both snakes and spiders^18, 36^. Similarly, in a visual search task, adult participants detected snakes more quickly or accurately than spiders^21, 22^. In conclusion, the human reaction to snakes and spiders does not seem directly comparable.

Another hypothesis suggests that fear and disgust of spiders is a generalization of fear and disgust of other disease-carrying invertebrates^33^. On the one hand, He et al.^37^ in their event-related potential (ERP) study demonstrated that there was no difference in attentional brain activation among spiders, wasps, bumblebees, or beetle stimuli, which pointed out to a high level of attentional generalization among stimuli. Contrarily, spiders and morphologically similar chelicerates were perceived specifically and differently compared to other invertebrates, including insects, based on subjective fear and disgust evaluation^30, 32, 33^. Generally, spiders are perceived specifically in subjective emotional evaluations and people do not generalize them to other arthropods, whereas in experimental measures of attentional response, the degree of generalization varies and requires further investigation. A similar hypothesis suggesting that fear of spiders is a generalized fear of scorpions was also not fully supported by subjectively perceived fear or disgust. Scorpions from this perspective form a separate, albeit sister category to spiders^30, 33^. Rudolfová et al.^38^ investigated spontaneous attentional bias toward scorpions and spiders in a cross-cultural eye-tracking experiment. The participants were presented with two picture stimuli at once in a free viewing task. Stronger attentional bias for scorpions as opposed to spiders was demonstrated in Somalis and a similar albeit weaker bias was also found in Czechs.

In the following eye-tracking study, we measured an attentional, behavioral, and emotional aspect of the reaction to snakes, spiders, crabs, and scorpions used as distractors to uncover a pattern of generalization among these stimuli. Physiological and psychological responses are often interlinked, and some physiological parameters are considered a good proxy for psychological processes^39^. For example, electrodermal activity can reflect subjective emotional arousal or significance of the animal stimuli^40, 41^, especially snakes^24^, and is thus a useful method in both basic and applied research^24, 42^. The term visual attention generally refers to a complex of cognitive operations that isolate the relevant information from the irrelevant one in a complicated visual scene^43^. In this study, the investigated animal was used as a task-irrelevant distractor. A similar design was previously used, for example, by Zsido et al.^44, 45^. We used two types of biases to create a distraction of visual attention devoted to a perceptual-cognitive task. The first bias comes from preferential attention paid to animate over inanimate objects^46, 47^, with threat-relevant animate stimuli (e.g. snakes, angry faces) being especially prioritized (see above). Another bias highly influencing visual attention is the central fixation bias which means that people looking at a picture pay more attention to its center^48, 49^. As our distractor image appeared in the center, the participants needed to exert an effort to shift their attention from the central stimulus and find the target.

An eye-tracking method is often combined with measuring reaction time by pressing a key or touching a screen^50–55^. The advantage of using parameters like reaction time is that it contains not only the aspect of exogenous attention but also behavioral adaptation and coping with potential threats that may present a distractor image, especially for people with a high level of specific fear of spiders^36, 56–61^. Pupil response reflects emotional reaction as well as a variety of other factors. The iris dilator muscle is controlled by the sympathetic nervous system which is activated during fear emotion and fight or flight reaction; its activation leads to pupil dilatation. Pupil dilatation may, thus, reflect general alertness^62, 63^ or it can be related to cognition^64–68^ and affective manipulation [Ref.^69–72^, reviewed in Ref.^62^]. From the cognitive perspective, pupil dilatates as a function of task difficulty (language-based^73^, visual search task^74^) and it can be used as a proxy for the individual amount of effort needed to accomplish the task^75^. On the other hand, the pupil constricts in response to increasing brightness and/or when the participant is close to finishing the task^76^. During our cognitive task, the respondents’ pupils might react by constriction (focus on the task, being close to finishing the task) or by dilatation increasing the pupil diameter while exploring the task as well as automatically searching for a potential danger (snake, scorpion, or spider). In general, attention, alertness, and emotional experience all increase the pupil diameter^62, 77^. Thus, we expect the pupil mean diameter and maximal pupil width to reflect these changes during our 5-s-long experiment.

In this paper, we focus on whether the distraction of attention by task-irrelevant animals is specific to the spider stimuli compared to other invertebrates (scorpions and crabs) in spider-fearful versus non-fearful participants. In general, if spider-scorpion or spider-invertebrate generalization occurs during the task, we should expect increasing levels of subjective emotional evaluation, reaction time, and attention to the distractor, along with increasing individual sensitivity to fear of spiders (increasing scores on the spider fear questionnaire). Specifically, we focused on three types of responses that should be influenced by sensitivity to fear of spiders: (1) The attentional response in a cognitive task impaired by the presence of a distractor – participants with high fear of spiders will be distracted specifically by the spider images leading to higher latency in fixating the true target in both the within-subject (compared to other animal stimuli) and between-subject comparison (compared to participants with low fear of spiders). (2) The attentional and behavioral response – the same effect would be manifested by a longer reaction time (pressing the response button). (3) The attentional, cognitive, and emotional psychophysiological pupil response—the higher the fear of the distractor animal and the higher the effort for cognitive and attentional load devoted to the task, the wider the maximal pupil size (mean size) of the participant while watching the presentation slide. We predict the same direction although of a smaller magnitude for the disgust emotional response.

## Results

### Attentional response in visual search task impaired by distractor

Latency in fixating the true target for the first time differed between the participants and between the target positions within the participant (both incorporated as random factors), which was reflected by the significant improvement of the model after the random factors were added (odds ratio = 1209.49, p < 0.001). Nonetheless, the attentional response (latency in fixating the true target for the first time) was significantly affected by only one investigated fixed factor (explanatory variable) – Age (nomDF/denDF = 1/103, F = 6.72, p = 0.011, intercept = 6.54, coefficient = 0.009). This result signifies that older participants fixated the true target later than younger participants. All other investigated fixed effects were successively reduced since their effect was not statistically significant: Gender – Age interaction (nominator degrees of freedom/denominator degrees of freedom (nomDF/denDF) = 1/100, F = 0.05, p = 0.832), Animal category – SPQ score interaction (nomDF/denDF = 3/3669, F = 2.16, p = 0.091), Animal category (nomDF/denDF = 3/3669, F = 0.55, p = 0.650), Gender (nomDF/denDF = 1/100, F = 1.53, p = 0.219), and the SPQ score (nomDF/denDF = 1/100, F = 3.56, p = 0.062). For the frequency of a number of fixations on each animal distractor category, see Supplementary Table S1 and Supplementary Figs. S1 and S2.

### Attentional and behavioral response (reaction time) in visual search task impaired by distractor

As hypothesized, the reaction time was greater for participants with higher SPQ scores in images with the spider and crab distractors but not with scorpion or snake distractors. Neither the participant’s gender, age, nor their interaction affected the reaction time hence these effects were successively reduced: Gender – Age interaction (nomDF/denDF = 1/100, F = 0.10, p = 0.754), Gender (nomDF/denDF = 1/100, F = 1.18, p = 0.281), and Age (nomDF/denDF = 1/100, F = 3.34, p = 0.070). In contrast, the animal category, SPQ score, and their interaction were kept in the final model: Animal (nomDF/denDF = 3/3416, F = 3.66, p = 0.012), SPQ score (nomDF/denDF = 1/100, F = 5.57, p = 0.020), and Animal – SPQ score interaction (nomDF/denDF = 3/3416, F = 4.95, p = 0.002). The odds ratio (assessed with a likelihood-ratio test) between the full and reduced model was 4.18, p = 0.245.

The intercept estimates reflect the reaction time of participants with a low fear of spiders (estimated reaction time means for SPQ score = 0). The animal categories intercepts are 6.87 for the crab, 6.95 for the scorpion, 6.97 for the snake, and 6.89 for the spider – the higher the number, the higher the distraction effect of the animal stimuli. The intercept for the snake was the highest, significantly higher than the one for the crab (p = 0.003) and spider (p = 0.012), but not the scorpion (p = 0.498) signifying that participants with low fear of spiders were distracted the most by snakes (and scorpions).

**Figure 1:**
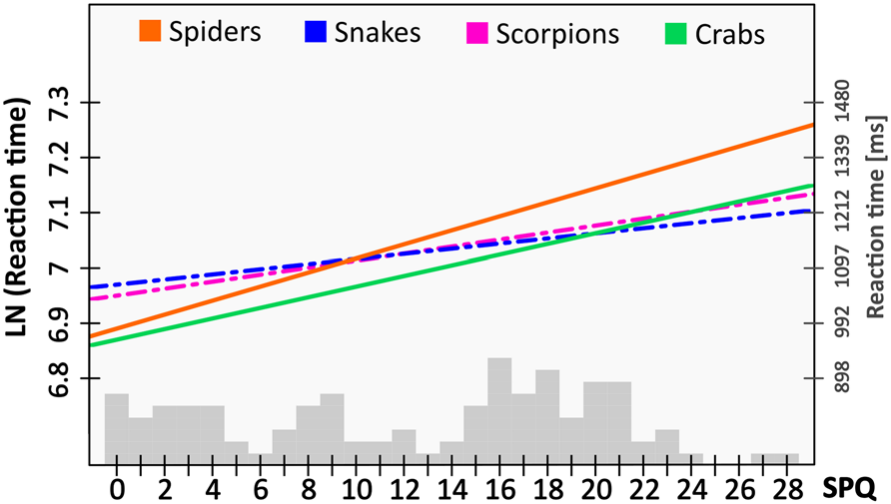
Results of the model for reaction time – behavioral response. The grey bars at the bottom represent the number of participants with respective spider questionnaire scores (SPQ). Note that the slopes of only solid lines are significantly different from zero, hence the effect of the SPQ score is significant only for spider and crab distractors.

The coefficient estimates reflect the reaction time of participants with increased fear of spiders (increased SPQ score). As predicted, the reaction time for the spider increased with a higher SPQ score (p = 0.001). This holds also for the crab (p = 0.011), but not the scorpion (p = 0.093), or the snake (p = 0.219). The slope coefficient for the spider is therefore significantly different from the one for the scorpion (p = 0.004) and snake (p < 0.001) but not for the crab (p = 0.129). This means that the participants most afraid of spiders were the most distracted by the spider and to some extent by crab stimuli. Contrarily, spider-fearful participants were not distracted by snake or scorpion stimuli more than participants with low fear of spiders, see Fig. 1. Note that all reported estimates were computed from natural logarithm-transformed values. Detailed results are shown in Table 1.

**Table 1:** Behavioral response—results of the model for reaction time.

Level/contrast	Intercept (DF = 3416)			SPQ score coefficient (DF = 103)		
	Est (95% CI)	t	p	Est (95% CI)	t	p
Crab	6.87 (6.77, 6.98)	–	–	0.01 (0.002, 0.02)	2.58	**0.011**
Scorpion	6.95 (6.84, 7.06)	–	–	0.006 (− 0.001,0.01)	1.70	0.093
Snake	6.97 (6.86, 7.07)	–	–	0.005 (− 0.003, 0.01)	1.24	0.219
Spider	6.89 (6.78, 7.00)	–	–	0.01 (0.005, 0.02)	4.00	**0.001**
Crab–Scorpion	− 0.07	− 2.31	**0.021**	0.003	1.52	0.129
Crab–Snake	− 0.10	− 2.97	**0.003**	0.005	2.28	**0.022**
Crab–Spider	− 0.01	− 0.43	0.667	− 0.003	− 1.40	0.161
Scorpion–Snake	− 0.02	− 0.68	0.498	0.002	0.78	0.436
Scorpion–Spider	0.06	1.86	0.062	− 0.006	− 2.92	**0.004**
Snake–Spider	0.08	2.53	**0.012**	− 0.008	− 3.67	** < 0.001**

For participants with a low fear of spiders, reaction time was the largest for snake and scorpion distractors. Sensitivity to fear of spiders affected only reaction time for spider and crab distractors. Hence, we found the greatest difference in reaction times between spider-scorpion and spider-snake in participants with various levels of spider fear. *SPQ score* spider questionnaire score, *DF* degrees of freedom, *Est* an estimate of the intercept/coefficient/contrast, *95% CI* 95% confidence interval, *t* t-value, *p* p-value, p-values < 0.025 are in bold. Note that all estimates were computed from natural logarithm-transformed values.

### Psychophysiological pupil response and subjective emotional evaluation of distractors

Regarding the pupil metrics, we found that participants’ maximal pupil size was larger when the animal distractor was rated as more fear-eliciting. Contrary to that, no effect of fear on mean pupil size nor of disgust on either response was found since the p-values did not reach the level of significance adjusted by Bonferroni correction (α = 0.0125). Results of the models for trial maximal pupil size: Fear – nomDF/denDF = 1/4094, F = 10.16, p = 0.001, intercept = 993.25, Fear coefficient = 3.054; Disgust – nomDF/denDF = 1/4094, F = 6.15, p = 0.013. Results of the models for trial mean pupil size: Fear – nomDF/denDF = 1/4094, F = 6.10, p = 0.014; Disgust – nomDF/denDF = 1/4094, F = 3.71, p = 0.054. For better comparability with the attentional and behavioral response (reaction time), we additionally computed a second model for trial maximal pupil size with Animal, SPQ-score, and their interaction as fixed factors. Similarly, to attentional and behavioral response, the interaction was highly significant: nomDF/denDF = 3/4089, F = 5.11, p = 0.002. Participants with a low fear of spiders had the largest pupil when a snake distractor was displayed, followed by crab, scorpion, and spider, even though only snake and spider distractors significantly differed from each other (p = 0.005). With a growing fear of spiders, the maximal pupil size grew more when spider distractors were displayed compared to crab (p = 0.004), scorpion (p = 0.002), and snake (p < 0.001) distractors; for detailed results see Supplementary Table S2.

**Figure 2:**
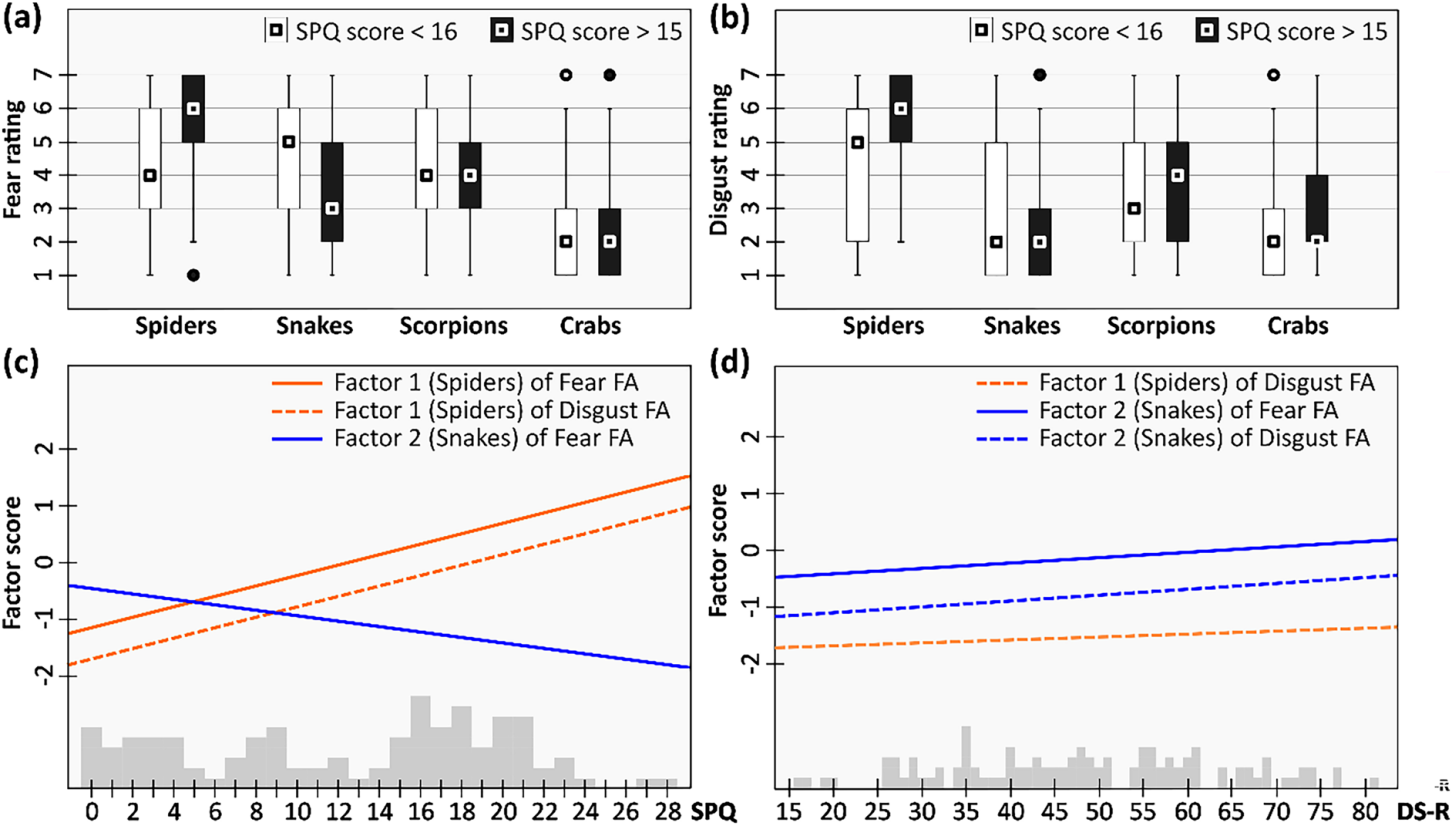
Results of the image rating. Box plots of fear (**a**) and disgust (**b**) ratings, and the effect of SPQ score (**c**) and DS-R score (**d**) on factors scores (rating of images); grey bars at the bottom represent the number of participants with respective scores. *SPQ* SPQ score – participant’s spider questionnaire score. *DS-R* participant’s disgust scale-revisited score.

Results of image ratings according to elicited fear and disgust are shown in Fig. 2a and b, respectively. Results of FAs for fear and disgust both revealed a simple structure of four factors with Factor 1 loaded by spider ratings, Factor 2 by snake, Factor 3 by crab, and Factor 4 by scorpion ratings. The rating of the antelope did not correlate with any of the factors. Detailed results of FAs are shown in Supplementary Table S3. In successive modeling, the effect of the tested variables (SPQ score, DS-R score, Gender, Age, and Gender – Age interaction) proved significant only in a few cases (in the following set of models, the α level adjusted by Bonferroni correction was 0.00625). Spiders were rated as more fear-eliciting by participants with a higher SPQ score (Factor 1 of Fear FA: SPQ score – degrees of freedom/residual degrees of freedom (DF/rDF) = 1/102, F = 86.56, p < 0.001; intercept = − 1.150, SPQ score coefficient = 0.092, adjusted R^2^ = 0.45). Similarly, they were rated as more disgust-eliciting by those with a higher SPQ and DS-R score (Factor 1 of Disgust FA: SPQ score – DF/rDF = 1/101, F = 104.95, p < 0.001; DS-R score – DF/rDF = 1/101, F = 27.15, p < 0.001; intercept = -1.702, SPQ score coefficient = 0.092, DS-R score coefficient = 0.012, adjusted R^2^ = 0.56).

On the other hand, snakes were rated as more fear-eliciting by participants with a lower SPQ and higher DS-R score (Factor 2 of Fear FA: SPQ score – DF/rDF = 1/101, F = 9.42, p = 0.003; DS-R score – DF/rDF = 1/101, F = 13.48, p < 0.001; intercept = -0.451, SPQ score coefficient = -0.048, DS-R score coefficient = 0.022, adjusted R^2^ = 0.17) and as more disgust-eliciting by people with a higher DS-R score (Factor 2 of Disgust FA: DS-R score – DF/rDF = 1/102, F = 15.55, p < 0.001; intercept = − 1.140, DS-R score coefficient = 0.024, adjusted R^2^ = 0.12). The rating of spiders or snakes was not affected by the participant’s gender, age, or their interaction. Neither the fear nor disgust rating of crabs was affected by any of the investigated variables. The disgust rating of scorpions seemed to be affected by the participant’s gender-age interaction but the effect disappeared when an insignificant term (DS-R score) was reduced from the model. The effect of SPQ and DS-R scores on factor scores (rating of images) is shown in Fig. 2c and d, respectively. Detailed results of modeling are shown in Supplementary Table S4.

Basic statistics of the latency in fixating the true target for the first time (attentional response), reaction time (behavioral response), and the mean and maximal pupil size (emotional response) can be found in Supplementary Table S5.

## Discussion

We found no difference in the disruption of visual attention measured by the first fixation of the target but a large difference in the attention connected with the behavioral response (reaction time) and emotional (maximal pupil size) of spider-fearful participants to images with spider distractors. As predicted, spider-fearful participants had a longer reaction time when there was a spider distractor compared to snake and scorpion distractors. Their attentional and behavioral response (reaction time) was also slower compared to the control group of non-fearful participants. Concerning our original predictions, the results of the pupillometric models were less conclusive. The maximal pupil size, but not the mean pupil size, was influenced by the subjective emotional evaluation of distracting pictures, which means that not only the attentional and cognitive component but probably also the participants’ emotional engagement was important during the task. Image ratings confirmed that the SPQ score (individual sensitivity to fear of spiders) well described the participants’ perception of spiders.

### Attentional response in visual search task impaired by distractor

We hypothesized that participants with a high fear of spiders would show higher latency in fixating the true target both compared to other animal stimuli and to participants with a low fear of spiders. However, we found no such difference as neither the SPQ score nor the animal image proved to have a significant effect on the latency of the true target's first fixation. Fear-eliciting or threatening stimuli have long been hypothesized to capture human attention. This was illustrated, for example, by Lundqvist & Öhman^78^ or Yorzinski et al.^1^. Originally, two mechanisms behind this phenomenon have been suggested. It could be that fear-eliciting stimuli attract the participant’s attention more easily, or that fear-eliciting stimuli hold the participant’s attention longer^79^. In recent years, a consensus seemed more inclined towards the latter mechanism^50, 80^, however, a recent meta-analysis suggests that both mechanisms can be relevant^81^. We could not directly test whether our data support either hypothesis. The participants were instructed to search for the target and look at it until it disappeared. They embraced this task, allowing for a robust analysis of attentional and behavioral responses to the target. Consequently, though, they paid very little attention to the animal distractors making it difficult to analyze animal fixation frequency, animal dwell time, or mean gaze duration on the animal because of low variability. Moreover, the central placement of animal distractors led to zero latency of the first gaze on the animal in almost all cases.

However, we cannot exclude the possibility that the tested parameter of latency in fixating the true target in the visual search task was mostly influenced by the cognitive aspect of the task, and thus by the individual performance. The significant effect of age supports this view. We found that younger participants fixated the true target on average faster than the older participants. Several specific phenomena could explain this result, for example, a slower onset of solving the task after the start of a trial, or less exposure to (experience with) computers, which could be expected in older rather than younger participants.

### Attentional and behavioral response (reaction time) in visual search task impaired by distractor

As we predicted, the spider-fearful participants’ behavioral response was slower in comparison to non-fearful participants, most probably because they were distracted by the spider images. Importantly, spider-fearful participants were specifically distracted by spider stimuli and not by scorpions or snakes (non-significant SPQ score coefficients, see Table 1 and Fig. 1). It has been previously shown that emotions can modulate attention toward a stimulus and facilitate its detection. In a visual search task, Soares et al.^50^ reported that participants found the animal they were afraid of faster than a non-feared but fear-relevant animal. Specifically, spider-fearful participants found spiders faster than snakes and conversely, snake-fearful participants found snakes faster than spiders. Flykt et al.^82^ additionally found that fearful participants pressed the response button harder when the target animal was their specifically feared animal. Even when the feared animal is not the target, it can affect attention. Miltner et al.^83^ found that the presence of task-irrelevant spider distractors slowed the detection of mushroom targets in spider-fearful participants. Our results, therefore, are in agreement with these studies.

In this study, we placed the distractor stimuli in the center of the slide and started a new trial only when the participant’s gaze was fixed on the center. Consequently, the participants were aware that one of the four animals would soon appear in the center of their visual field. While non-fearful participants did not differentiate the spider from other animals in a way that would affect their “success” in solving the task, spider-fearful participants were selectively affected by spider and crab distractors. This suggests that spider-fearful participants were alert and anticipated the appearance of frightening stimuli. The importance of expectations was previously investigated by Devue et al.^84^ who reported that spider-fearful participants performed poorly in trials with potential spider stimuli but similarly well as the control group when they knew no spider could be expected. Higher alertness when potentially dangerous stimuli are suspected to appear is crucial for the quick activation of an appropriate physiological and behavioral response. Even though high fear of spiders and/or arachnophobia might not be adaptive themselves and are also often consciously considered “nonsensical”, they activate highly adaptive pathways.

Surprisingly, although the crabs were not subjectively rated as fear- or disgust-eliciting by the spider-fearful participants, they distracted them similarly to spiders. In comparison with crab stimuli, the distracting effect of snakes or scorpions on reaction time did not resemble the trend of increasing effectivity to distract more sensitive respondents that we found for spiders (see Fig. 1). We account for this due to the high morphological similarity between crabs and spiders (specifically those selected here as stimuli) which made them easy to be confused by just a glance. From an adaptive point of view, the threshold for what is and what is not a feared stimulus has to change to secure as few false-negative responses (overlooked real signals) as possible when the participant’s attention is directed toward the animal-unrelated task. This, however, can only be done at the expense of false positives (responses to incorrect stimuli). Still, human attention is very fine-tuned for the spider stimuli because scorpions – similar in appearance and biologically close relatives to spiders – did not affect the success of solving the task. A similar result was also shown by New & German^35^ where participants’ responses to spiders and house flies differed in an inattentional blindness task.

To the best of our knowledge, the current study is the first one that utilized scorpions as distractor stimuli and compared them to snakes. Interestingly, the scorpion distractors’ effect on the attentional and behavioral response did not resemble the effect of spiders but rather snake distractors in our dataset. This was quite surprising for snake stimuli because snakes have long been considered a special fear stimulus. This notion was supported by several studies (see “Introduction”) including the eye-tracking ones that reported faster or more accurate detection of snakes (e.g. see Ref.^2^). Later, this view was questioned. It was pointed out that snake detection could have been facilitated simply because they were compared to flowers and mushrooms and not to other animals^85^. Later again though, the snake’s specificity was confirmed with regards to its distractor properties or when detection took place under challenging setups^5, 22^. Here, all the stimuli worked as effective distractors but each for a different group of participants. For participants with a low fear of spiders, both snakes and scorpions triggered a behavioral response more than the control, fear-irrelevant stimulus – the crab. This supports the hypothesis of the scorpion as the evolutionary fear-relevant stimulus similar to the snake (see Ref.^30^ for details). However, the distractor effect of crabs increased with the participant’s SPQ score inevitably influencing the estimated crab-snake and crab-scorpion differences. Although we find the similarity between snakes and scorpions highly interesting, more research is certainly needed.

As all the distractors are effective, it seems that several types of generalization might occur: (1) Either the generalization is based on function, and in this case, associated emotions seem adaptive (i.e. both snakes and scorpions are objectively fear-relevant, therefore a fear-mediated behavioral response is generally advantageous). (2) Alternatively, the generalization is based on physical features (as we suspect was the case for spiders and crabs) which may be advantageous, disadvantageous, or neither of those depending on the situation. It is worth pointing out, though, that from the evolutionary perspective, it is also adaptive to allow some level of mistakes^86^. (3) According to New et al.^46^, all animal and human stimuli generally attract attention and affect the reaction time in the same way. This remains questionable as we found the effect of stimulus animal category on the reaction time and differences in subjective emotional evaluation of these animal categories. However, we were not able to fully elucidate the small differences between them in our task.

### Psychophysiological pupil response and subjective emotional evaluation of distractors

Lastly, we predicted that the participants’ mean and maximal pupil size would be wider when watching the presentation slide with subjectively more feared animal distractors. We found this effect for the maximal but not the mean pupil size. Further, we did not find any effect of perceived disgust on maximal or mean pupil size. It is generally agreed that emotional stimuli affect the pupil diameter. Both positive and negative emotional stimuli are usually associated with pupil dilation^87^, however, pupil constriction was also noted in some cases. Specifically, pupil constriction was usually found in studies focusing on disgust^88^, which involves the activation of the parasympathetic nervous system^89^. On the contrary, fear elicits a sympathetic activation, leading to pupil dilation^90^. In this regard, the spider is a particular stimulus because it evokes both strong fear and disgust, even though fear is usually stronger^33^; see Fig. 2a,b). Additionally, it was also shown that pupil dilation increased with a cognitive effort to solve the task. Emotions and cognition can also interact^91^. In other words, pupil dilation might be the result of several processes and the extent to which each may affect the pupil diameter remains unclear. In this study, we mainly focused on the attentional and behavioral response and pupillometry was rather supplemental, hence the feasibility of some analyses was limited. Additional cautiousness should be executed when interpreting the results because while EyeLink1000 offers pupillometric characteristics, its primary purpose is different.

Based on the analysis of image ratings, all images of one type of animal were perceived similarly to each other but as distinct from images of different types of animals. This confirmed that the images were appropriately selected to represent one category and that their grouping into one factor of four levels in statistical modeling was justified. Unsurprisingly but importantly, we also confirmed that participants with a higher SPQ score rated spiders as more fear-eliciting and disgust-eliciting than participants with a lower SPQ score (see Fig. 2). This means that a subjective animal rating, a semi-objective SPQ score, and an objective behavioral response are in good concordance and give similar results. In research on the effect of fear elicited by spiders, participants are often divided into two groups which represent two extremes, meaning participants with medium fear or ambiguous emotions (the middle of the scale) are not represented [e.g., 85]. This approach has several advantages, e.g. thanks to the higher difference between the participants, smaller effects can be detected. However, we find relatively even coverage along the whole SPQ scale useful (see, Figs. 1 and 2c), as well, since it allows us to access the value of behavioral response for any SPQ score (within the included range).

We further found that fear of snakes declined with higher SPQ scores (Fig. 2c). This means that, in our sample, participants who were not afraid of spiders tended to fear snakes more than spider-fearful participants. However, we do not think that this result should be generalized to the population level as it is most probably a by-product of the experimental design (see also Ref.^28^). On the one hand, a high fear rating of snake stimuli should be expected since they were all highly venomous vipers^10, 24, 92^. On the other hand, to spider-fearful participants, spiders are the most salient stimuli no matter the objective dangerousness of others, and therefore snakes are rated as less frightening in comparison. Combined, this led to spider-fearful participants being seemingly less likely to fear snakes than the control group. Our explanation is also supported by the frequency of self-reported fear of snakes (yes or no answer to the question: “Are you afraid of snakes more than you consider usual?”). Ten (out of 55) participants from the control group and 13 (out of 50) spider-fearful participants reported being (also) afraid of snakes – a rather similar proportion.

Finally, we found that fear of snakes and disgust of spiders and snakes increased with an increasing DS-R score, but the relationship was rather weak (Fig. 2d). This is in concordance with Arrindell et al.^93^ who also found that disgust sensitivity held only a little predictive value about animal fears.

## Conclusions

In this study, we replicated the general experimental design of several previous studies (i.e. the task-irrelevant distractor design^44, 80, 83^) and found comparable results. In short, subjectively feared spiders distracted participants from solving the task to a greater extent than objectively more fear-relevant snakes and scorpions. However, we also modified the design in numerous important and new ways. First, we recruited participants covering quite evenly almost the full range of the SPQ scale. Second, we used rather large animal images (the distractors) and placed them in the center of the visual field to ensure that participants would gaze at them and enhance the potential distractor effect. Third, we added two new uninvestigated animal stimuli – a crab and a scorpion.

The overall fear response to spiders as distractors was quite specific. Spider pictures distracted the attention and behavioral reaction of spider-fearful participants in the visual search task more than pictures of snakes and scorpions. Individual sensitivity to fear of spiders (measured by the SPQ) also influenced subjective emotional image ratings. People with a higher fear of spiders rated spiders as more fearful and snakes as less fearful than people with low SPQ scores. Interestingly, emotional subjective ratings of pictures of other invertebrates (scorpions or crabs) were not related to individual sensitivity to fear of spiders. The subjective evaluation of pictures according to fear was also reflected in the pupillary reaction: the higher the fear image ratings the higher the pupillary responses. This result was more general and did not apply exclusively to ratings of spiders by spider-fearful participants. Thus, we showed that some parameters of pupillary response reflected not only the attentional and cognitive components but also the emotional response to the stimuli. Taken together, we supported the view of emotions affecting attention as an important mediator in the behavioral and physiological response.

Interestingly, the effect of two previously uninvestigated distractors (crabs and scorpions) proved to be very different from each other – while the effect of the crab quite closely resembled the effect of the spider distractor, the scorpion was more similar to the snake distractor. We hypothesize that spider-fearful participants might have mistaken the crab for a spider because of their high morphological similarity in this stimulus set. In other words, spider-fearful participants generalized their reactions from spiders to crabs based on their shared physical characteristics. Contrary, participants with no fear of spiders were distracted the most by snakes and scorpions. No difference between snake and scorpion distractors was found supporting the notion that scorpions are also prioritized, evolutionary relevant stimuli. However, we did not confirm the generalization of the fear response from scorpions to spiders and thus we cannot support the evolutionary scenario with scorpions being the prototype stimuli for the evolution of attentional and fear response to the spiders.

## Methods

### Participants

A total number of 114 participants were originally recruited for the experiment. Of these, 50 participants were undergraduate Czech or Erasmus students who participated for a credit in the Ethology and Sociobiology course. The remaining 64 participants were recruited from our database of Czech research volunteers based on their scores on the Spider Questionnaire (SPQ^94^). Seven participants finished the eye-tracking experiment but chose not to follow through with the image rating (two participants) or completion of the questionnaires (five participants), hence their data were excluded from the analyses. Due to technical difficulties during the eye-tracking data extraction, the data of two additional participants had to be excluded. Therefore, the final sample consisted of 105 participants, 84 women, and 21 men (mean age 25.70 years, range 18–49). Based on their SPQ scores, 55 participants (38 women) had none to moderate fear of spiders (SPQ < 16), while the remaining 50 participants (46 women) had high or very high fear of spiders (SPQ ≥ 16^28^). All the participants had normal or corrected-to-normal vision.

### Stimuli

**Figure 3:**
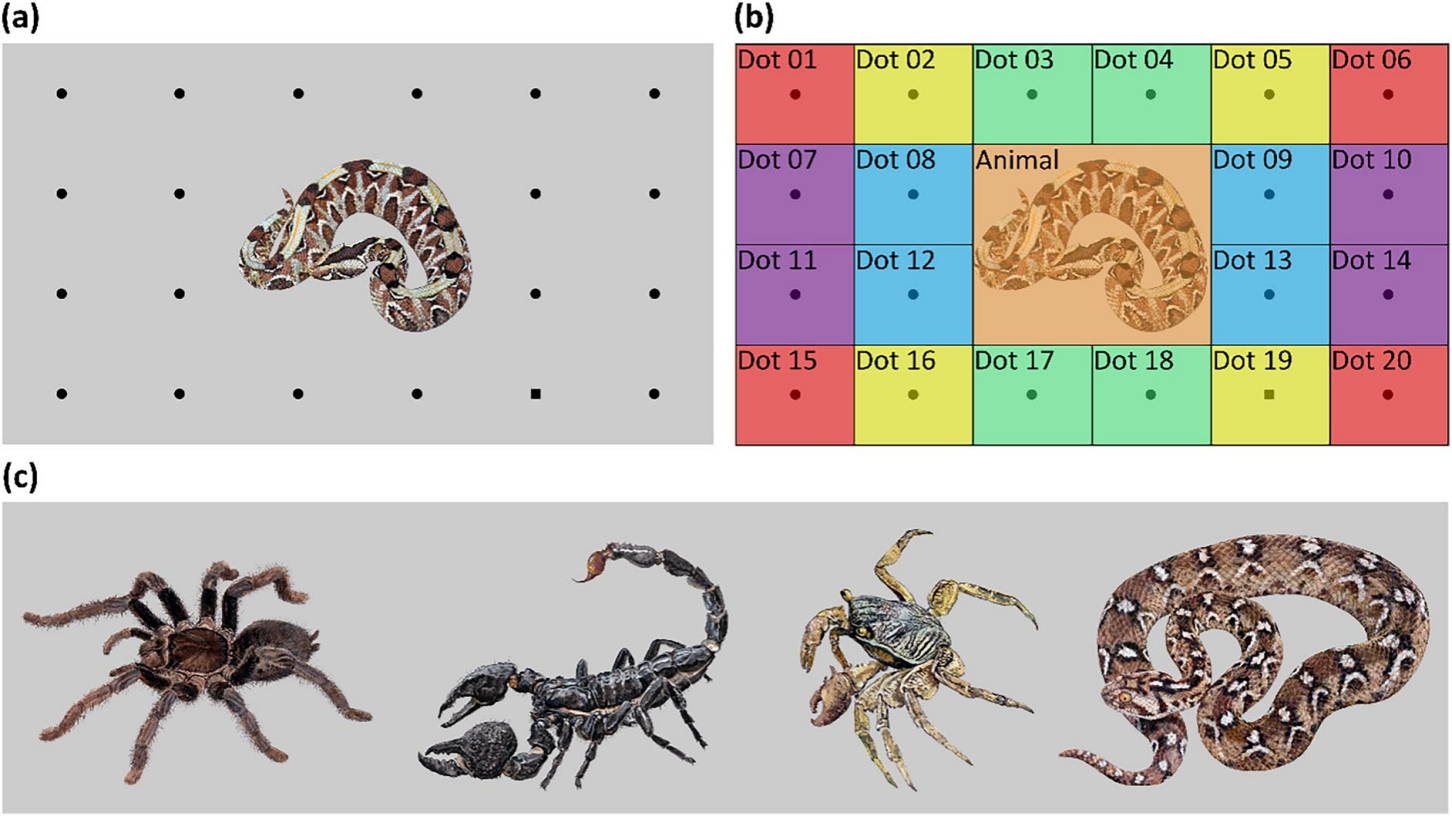
An example of the experimental slide as shown to the participants (**a**) and as overlayed by areas of interest (**b**), and additional examples of animal distractors – one for each animal group (**c**). In the experimental slide example, the distractor is a snake, the false targets are on positions 1–18, and 20, and the true target is on position 19 (area of interest ‘Dot 19’). Target positions of the same type (level) are shown in the same color. The closest to the slide center are those in green (approx. 435 px), followed by the blue (approx. 498 px), yellow (approx. 627 px), purple (approx. 811 px), and red (approx. 897 px) ones (the distance was measured from the center of the dot to the center of the slide). Images in (**c**) are not in scale. Due to copyright restrictions, animal photos presented to the participants have been replaced by illustrations highly authentic to the original photo.

Each eye-tracking stimulus slide (1920 × 1080 pixels) was a color image of an animal (the distractor) surrounded by 19 dots (false targets) and 1 square (true target) placed on a neutral 20% grey background (see Fig. 3). The animal images were sourced from the internet or our database. Criteria for the selection were good resolution of the image, full-body depiction of the animal, and “neutral” body posture. The animal’s original background was cut off and it was placed and sized to fit into the central area of approx. 640 × 540 px. Animal images were of four categories – crabs, scorpions, snakes, and spiders – each represented by five different species. The dots had a diameter of 29 px and the side length of the squares was 26 px making the shapes comparable in size (their area, as well as width and height); all dots and squares were black. They were placed in a 6 by 4 grid with four central positions left out for the animal image. Target positions were of five types (levels) based on their distance from the center. Positions 3, 4, 17, and 18 were the closest to the center while positions 1, 6, 15, and 20 were the most distant (see Fig. 3b). A total of 20 unique experimental slides were created using each combination of the animal category and true target position level just once. Additionally, images of antelopes in the same layout were used to create five practice stimuli.

For the eye-tracking analysis, 21 unique areas of interest (AOI) were defined. In the center, there was the animal AOI (640 × 540 px in size). Surrounding each of the 20 grid positions, there were the dot 01–20 AOIs (each 320 × 270 px in size). The AOIs did not overlap and covered the whole slide (see Fig. 3b).

### Procedure

First, each participant was briefed about the subsequent tasks and gave written consent for his/her participation in the research. The subsequent eye-tracking experiment consisted of 5 practice trials followed by 40 experimental trials. Then, they were instructed to rate the presented stimuli based on the level of elicited fear and disgust. Lastly, they completed two questionnaires: the Spider Questionnaire (SPQ; Czech translation by Polák et al.^28^) and the Disgust Scale-Revised (DS-R; Czech translation by Polák et al.^95^). If the participants already completed these questionnaires as a part of previous research (as was the case for all non-student and some student participants), this last step was skipped. All instructions and questionnaires were given in Czech to Czech and Slovak participants and in English to Erasmus student participants (originally from 8 other European countries).

### Eye-tracking

Eye movements were recorded using the EyeLink1000 eye-tracking device; for measuring the reaction times, participants answered using a response box. The participants were seated in front of a 19-inch screen (Full HD resolution, refresh rate 60 Hz) and their head was fixed using a chinrest at a distance of 70 cm from the screen. The central animal distractor (the width and height, respectively) subtended 11.15° and 10.61° of the visual angle (corresponding values for the whole screen were 32.65° and 21.04°, respectively). In the beginning, they answered four questions: age, gender, handedness, and country of origin. Next, the device was calibrated using a standard nine-point calibration procedure, involving the fixation of ten target crosses presented on the computer screen positioned on a three-by-three grid (the first and last target cross was presented in the center). The calibration was subsequently validated with the average allowed error ≤ 0.5° and maximal allowed error ≤ 1° of the visual angle. If the error was higher, the device was adjusted, and calibration and validation were repeated. After successful validation, the experimental task was explained in detail. Participants were instructed to find the square as fast as possible, signal the finding by pressing any button of the response box, and keep their gaze on the square until the whole stimulus slide disappeared. Slides with stimuli were presented for 5 s comprising a trial. The stimuli slides alternated with slides presenting a target cross in the center; the next stimulus slide was presented right after the participant fixated on the target cross (the target cross slides were hence presented for variable periods but generally for under a second). Firstly, five practice stimuli were presented to the participants to get familiar with the response box and the task. Following the practice stimuli, they were offered an opportunity to ask questions. Next, all 20 stimuli were presented in two series, each time in a random order (different for each participant). In total, the experiment consisted of 5 practice trials and 40 experimental trials (10 trials per animal distractor category). The experimental setup was designed, and the stimuli were presented using the SR-Research Experiment Builder.

Using the DataViewer (SR-Research), we extracted the following variables. For the whole trial: reaction time (RT), trial dwell time, trial fixation count, median fixation duration, blink count, minimal pupil size, maximal pupil size, mean pupil size, saccade count, the total number of visited areas of interest, and sequence of visited areas of interest. For each area of interest (AOI), we additionally extracted AOI dwell time, AOI fixation count, and AOI latency of the first, second, and third fixation.

### Rating of images

Participants rated 21 animal images in total – 20 experimental images and one image of an antelope used in the practice trial. We uploaded the images to a special web application available at www.krasazvirat.cz. Each image was rated on perceived fear and disgust on a seven-point Likert scale (1 corresponded to no fear/disgust, 7 corresponded to very strong fear/disgust). The animals were rated on both emotions at once, and the order of the images was random and different for each participant. Before the rating, each participant filled in a short questionnaire regarding their age, gender, type, and level of education, and whether they considered themselves to be afraid of any animal(s) more than they perceived as usual.

### Selection of investigated parameters

Multiple parameters like the total number of fixations, total fixation duration, latency to the first fixation, or number of blinks are commonly used for measuring attention (reviewed in Skaramagkas et al.^96^). For example, all participants looked first at spiders (before butterflies, cats, and dogs), however, spider-phobic participants quickly disentangled their gaze from the phobic stimulus^97^. LoBue et al.^98^ used the number of first fixations in two simultaneously presented pictures (snake vs. frogs, angry vs. neutral faces, happy vs. neutral faces) and latency to the first fixation of a probe in their adapted dot probe task for infants. Infants fixated snakes more often than frogs and fixated faster the probes that appeared instead of snakes. Malcolm and Henderson^99^ reported a similar cognitive aspect in their task where participants were able to search for a target even if it was a non-salient object in the scene containing distracting highly salient objects. For this reason, we choose latency to the first fixation of the target as a parameter of attentional and cognitive effort devoted by the individual respondent to the task in the condition of attentional distraction by the central animal image. A similar idea was used in a previous study by Soares et al.^36^ where animal pictures served as distractors in the center of a screen and a group of letters was a task-relevant stimulus. Regarding the pupillometric parameters, we used the absolute pupil diameter as it may be an appropriate measure to compare different blocks of the task^100^ represented in our case by different categories of distractors.

As additional factors, we also included the participants’ gender and age. It has been shown that women are affected twice as often by animal phobias^101, 102^ and they fear the phobic stimulus more intensively than men^32, 103^. Women also score higher on fear of snakes^104^, spiders^105^, and disgust propensity^95^. They also rate pictures higher according to perceived fear or disgust^11, 33, 92^. The effect of age (decreasing emotional sensitivity with age) is less prominent than the gender effect of gender but should still be taken into account^11, 28, 106^. The younger respondents could also have faster reaction time owing to more experience with electronic devices. Thus, we included gender and age as supplementary factors in our analyses.

### Statistical analysis

Firstly, we analyzed data accessed from the eye-tracking experiment; only data from experimental trials were analyzed. We used linear mixed-effects models (LMM) and the maximum-likelihood method as implemented in the software RStudio^107, 108^, package nlme^109^. For the analysis of the attentional and emotional response, the full dataset was utilized (all trials were included). However, for the analysis of the behavioral response, the dataset was curated as follows. (1) We excluded all trials where the reaction time was shorter or the same as the latency in fixating the true target for the first time; there were 253 such trials. (2) If the participant did not press the response button during the trial, we set the reaction time to 5000 ms; there was one such trial.

To test the attention and behavioral response-related hypotheses, we successively built LMM with latency in fixating the true target for the first time and the reaction time as response variables, and the Animal category, SPQ score, and Animal category – SPQ score interaction as fixed effects. We also checked for the effect of Gender, Age, and Gender – Age interaction. In both models, we used the participant’s ID and target position (5 levels, see Fig. 3b) within the participant’s ID as the random effect. As neither of the response variables had a normal distribution, we used a natural logarithm transformation. To avoid false-positive effects because of testing multiple variables from one dataset, we employed the Bonferroni correction; we tested two eye-movement-related variables (latency in fixating the true target for the first time, and reaction time), thus we set α = 0.05/2 = 0.025 for these two variables. In the emotional response set of LMM models, mean and maximal pupil size were response variables, while fear and disgust respectively were used as fixed effects and the participant’s ID was used as a random effect. Since we tested four hypotheses related to pupil automatic reaction, we again implemented Bonferroni correction and set α = 0.05/4 = 0.0125 for these four models. We utilized a top-down approach and successively reduced fixed effects that did not prove significant. The likelihood ratio test and the Akaike information criterion (AIC) were employed to compare the reduced models and their corresponding full models. The first method supported the same goodness of fit of both models (the full one and the reduced one), while the AIC suggested the reduced models were better because they were more parsimonious.

Another potentially interesting variable – the number of fixations on the distractor animal – could not have been analyzed because of low variability. The participants were instructed to search for the target and look at it until it disappeared. In 2054 cases out of 4200, there was only one fixation on the animal at the beginning of the trial and once the participants started to solve the task, they indeed did not look at the animal. See Supplement S4 for the histogram and frequency table of the number of fixations on the distractor animal.

Secondly, we analyzed the rating of images. To avoid multiple testing, we performed factor analysis (FA) separately for fear and disgust ratings. We employed the principal component method, extracted four factors, and used the Varimax normalized rotation. For each factor, we extracted factor scores and hence defined eight new variables (factors). We used linear models (LM) as implemented in RStudio, to test the effect of SPQ score and DS-R score, as well as Gender, Age, and Gender – Age interaction on the factor scores (participants’ rating of images, eight models in total). Similarly to LMM, variables and their interactions that did not prove significant were successively reduced. We again implemented the Bonferroni correction and set α = 0.05/8 = 0.00625 to avoid reporting false-positive effects and compared the residual sums of squares of full and corresponding reduced models by analysis of variance.

### Ethical note

All experimental protocols were approved by the Ethics Commission of the National Institute of Mental Health (approval no. 117/18, granted on 28 March 2018). The authors also confirm that all experiments were performed following relevant guidelines and regulations (such as the Declaration of Helsinki). Written informed consent was obtained from all participants included in the study.

### Supplementary Information

Supplementary Information.

## Data Availability

All data generated or analyzed during this study are included in the Supplementary Information files (Supplementary Table S6). For stimuli slides, please contact the corresponding author.
